# Prediction of postoperative hypoglycemia in patients with pheochromocytoma resection: a retrospective study

**DOI:** 10.1186/s12893-026-03734-1

**Published:** 2026-04-15

**Authors:** Shengwen Song, Jianrong Li, Lihua Chu, Xiangming Fang

**Affiliations:** Department of Anesthesiology and Intensive Care, The First Affiliated Hospital, Zhejiang University School of Medicine, Hangzhou, Zhejiang China

**Keywords:** Pheochromocytoma resection, Postoperative hypoglycemia, Plasma metanephrine, Beta blocker, Nomogram

## Abstract

**Background:**

Hypoglycemia after pheochromocytoma resection is one of the most common complications. The factors predicting postoperative hypoglycemia are not well established. The objective of this study was to develop a model to predict hypoglycemia in patients after pheochromocytoma resection.

**Methods:**

This single-center retrospective study enrolled adult patients who underwent pheochromocytoma resection between September 2019 and August 2025. Logistic regression identified risk factors for postoperative hypoglycemia, based on which a nomogram model was developed. Discrimination and calibration analyses were conducted to assess the performance of the model.

**Results:**

A total of 114 patients were enrolled in the final analysis. The median age was 56.5 years, and 53.5% were females. The overall incidence of hypoglycemia after pheochromocytoma resection was 27.2%. The length of hospital stay after surgery was longer in patients with hypoglycemia than those without (*P* = 0.020). In the multivariable logistic regression analysis, patients who developed hypoglycemia were younger (odds ratio [OR] = 0.95, 95% confidence interval [CI] 0.92 to 0.99, *P* = 0.015), and were more likely to have elevated preoperative plasma metanephrine level (OR = 7.73, 95% CI 2.00 to 29.85, *P* = 0.003) and to receive beta blocker intraoperatively (OR = 3.67, 95% CI 1.40 to 9.57, *P* = 0.008). The nomogram model showed good discrimination (area under receiver operating characteristic curve: 0.798, 95% CI: 0.714 to 0.882) and calibration (Hosmer-Lemeshow test *P* = 0.720) for predicting postoperative hypoglycemia.

**Conclusions:**

Hypoglycemia was common after pheochromocytoma resection and was associated with poor prognosis. The developed nomogram model incorporating age, elevated preoperative plasma metanephrine level, and intraoperative beta-blocker use can effectively predict postoperative hypoglycemia in patients undergoing pheochromocytoma surgery.

**Clinical trial number:**

Not applicable.

**Supplementary Information:**

The online version contains supplementary material available at 10.1186/s12893-026-03734-1.

## Background

Pheochromocytomas are rare neuroendocrine tumors with the reported incidence rate approximately 1.9 cases per million person-years [1]. Pheochromocytomas are characterized by uncontrolled catecholamine production, for which surgical resection remains the cornerstone of curative therapy [2–5]. However, the very success of tumor removal precipitates a profound endocrine crisis: the abrupt cessation of hormone secretion can trigger a cascade of life-threatening postoperative complications [6]. Profound hypotension is a well-recognized and routinely anticipated challenge in the post-resection period, while post-excisional hypoglycemia represents a more insidious and underappreciated threat [7]. Patients with hypoglycemia after pheochromocytoma resection often present with nonspecific symptom, including fatigue, or altered mental status. These nonspecific presentations frequently lead to a dangerous delay in the diagnosis and treatment of hypoglycemia, which may lead to severe neurological injury and deteriorate clinical outcomes [8, 9].

The ability to pre-emptively identify patients at high risk for postoperative hypoglycemia is therefore a paramount concern for clinicians to ensure a safe postoperative transition in patients who underwent pheochromocytoma resection. Current strategies for risk assessment remain rudimentary, relying predominantly on static preoperative variables such as baseline catecholamine levels, and tumor size [8, 10, 11]. While informative, these snapshots of tumor biology fail to capture the dynamic physiologic stress of the operation itself. The degree of intraoperative hemodynamic lability and the requirement for vasoactive drugs directly reflect the magnitude of catecholamine surge [12, 13]. Thus, they may serve as quantifiable intraoperative biomarkers that predict the subsequent postoperative metabolic dysregulation and hypoglycemia.

Therefore, this study aimed to develop and validate a model that integrates preoperative and intraoperative parameters to predict hypoglycemia in patients undergoing pheochromocytoma resection. Our objective is to provide a practical, physiology-based tool to enable clinicians to stratify risk proactively, facilitating targeted glucose monitoring and early intervention for the most vulnerable patients in the immediate postoperative period.

## Methods

### Study design and setting

This single-center, retrospective study was conducted at the First Affiliated Hospital, Zhejiang University, School of Medicine. The study protocol was approved by the Research Ethics Committee of First Affiliated Hospital of Zhejiang University School of Medicine (Approval Number: 2026B-0031), and the requirement for written informed consent was waived due to the retrospective nature of the investigation. This study was reported after the Strengthening the Reporting of Observational studies in Epidemiology (STROBE) guideline. We screened all adult patients who underwent surgical resection for pheochromocytoma in the operating room between September 1, 2019, and August 31, 2025.

### Patient selection

The inclusion criteria were adult patients (≥ 18 years) who underwent robotic or laparoscopic adrenalectomy. All enrolled patients were diagnosed with pheochromocytoma based on postoperative histopathological examination. Patients were identified from the hospital’s electronic medical records system.

Exclusion criteria were applied as follows: (1) Diagnosis of pheochromocytoma as part of a known hereditary syndrome to minimize pathophysiological confounding factors (e.g., Multiple Endocrine Neoplasia types 2A or 2B, von Hippel-Lindau disease, or Neurofibromatosis type 1); (2) Absence of invasive arterial blood pressure monitoring throughout the surgical procedure, which was essential for the acquisition of high-fidelity, beat-to-beat hemodynamic data; (3) Cases where the planned tumor resection was not completed (e.g., due to unresectable disease, intraoperative decision change, or conversion to an open procedure from a minimally invasive approach where such change was not part of the original plan); (4) Incomplete or inaccessible perioperative medical records preventing reliable extraction of key study variables.

### Anesthetic management and hemodynamic monitoring

This retrospective study analyzed the intraoperative management as documented in the anesthesia records. All patients underwent elective resection under general anesthesia with endotracheal intubation, which was maintained with either volatile inhalational or total intravenous anesthesia at the discretion of the attending anesthesiologist. Invasive arterial pressure monitoring was employed to ensure the continuous, high-fidelity acquisition of systolic blood pressure (SBP), diastolic blood pressure (DBP) and heart rate (HR) data. The placement of an arterial line was a prerequisite for study inclusion to allow for precise beat-to-beat hemodynamic tracking. All SBP, DBP and HR values, automatically recorded by the anesthesia monitoring system (GE Healthcare, Helsinki, Finland) at 5-minute intervals, formed the primary hemodynamic dataset. Hemodynamic management, including the use of vasoactive drugs to treat hypertension (alpha blocker), hypotension (e.g., phenylephrine, norepinephrine), or tachycardia (beta blocker), was guided by standard institutional practices and the clinical judgment of the anesthesia team. After surgical procedure, the patients would be transferred to the post-anesthesia care unit or intensive care unit depended on clinical decision.

### Data collection and calculation of hemodynamic variability

Data were collected from patients’ electronic medical records. The extracted parameters included demographic data (age, sex, body mass index (BMI), medical history, drug history), laboratory data (blood glucose, plasma metanephrine level, plasma normetanephrine level, hematocrit, location and size of the tumor), anesthesia and surgical related data and clinical outcomes (length of stay after surgery and hospitalization costs).

Hemodynamic parameters recorded intraoperatively by the anesthesia monitoring system were extracted and imported into Microsoft Excel (Microsoft office 2019, Washington, USA). The data collection period commenced 5 min before the induction of general anesthesia and concluded upon the end of surgery. Hemodynamic variability was assessed as the primary metric of intraoperative blood pressure and heart rate stability. For each patient, we quantified this variability using the standard deviation of all intraoperative measurements recorded at 5-minute intervals [14]. SBP Variability: Calculated as the standard deviation of all individual intraoperative SBP values. DBP Variability: Calculated as the standard deviation of all individual intraoperative DBP values. HR Variability: Calculated as the standard deviation of all individual intraoperative HR values. Perioperative systolic blood pressure variation (ΔSBP): calculated as the difference between the maximum and minimum systolic blood pressure recordings during surgery. These indices reflect the dispersion of intraoperative readings around the patient-specific mean, providing a measure of intraoperative hemodynamic instability.

### Outcomes

The primary endpoint of this study was the incidence of postoperative hypoglycemia within the first 24 h after surgery.

Blood glucose monitoring was performed according to standard clinical practice. In routine postoperative care, blood glucose was measured on the first postoperative morning as part of standard laboratory testing. In addition, glucose levels were not assessed at predetermined hourly or fixed intervals. Instead, glucose measurements would be triggered by the onset of clinical symptoms suggestive of hypoglycemia (e.g., diaphoresis, tremors, tachycardia, or altered mental status) or as part of routine clinical assessment when deemed necessary by the treating physicians [15].

The observation period commenced upon the patient’s admission to the post-anesthesia care unit or the intensive care unit and extended for 24 h postoperatively. An episode of hypoglycemia was defined as any documented blood glucose measurement of ≤ 70 mg/dL during this 24-hour window. Furthermore, the administration of intravenous glucose for the treatment of neurogenic symptoms clinically attributed to a sudden drop in blood glucose was also considered as meeting the primary endpoint, even in the absence of a contemporaneous glucose reading ≤ 70 mg/dL [16].

The secondary outcomes include postoperative length of hospital stay and hospitalization costs.

### Sample size estimation

The incidence of postoperative hypoglycemia following pheochromocytoma resection is reported to be approximately 45% in a previous study [17]. Based on the principle of having at least 10 outcome events per variable in multivariate regression analysis [18], and considering the 5 candidate factors identified in prior studies [9], a final sample size of total 111 patients was required.

### Statistical analysis

The quantitative data were tested for distribution using the Shapiro-Wilk method. Normally distributed continuous variables were described as mean and standard deviation (SD) and non-normally distributed variables were described as median and interquartile range [IQR]. Categorical variables were described in terms of the number (percentage). Quantitative data were compared using the independent t test or the independent sample Mann–Whitney U test depending on the distribution. The differences among categorical variables were tested using the Fisher’s exact test or chi-square test.

The Variance Inflation Factor was calculated based on the linear regression equation to assess whether multicollinearity exists among the independent variables. Univariate analyses were first performed to identify possible covariates. Variables with *P* < 0.05 were subsequently entered into the multivariable logistic regression model using the forward LR stepwise approach, in which the presence of postoperative hypoglycemia was the dependent variable. Potential predictors were sequentially removed if this exclusion did not result in a significant change in the log-likelihood ratio test. The results of the final multivariable logistic regression model were expressed as odds ratios (ORs) with corresponding 95% confidence intervals (CIs).

A Nomogram was constructed based on identified independent factors. The performance of the prediction model was evaluated using receiver operating characteristic curves (ROC) and area under the ROC curve (AUC). To assess the consistency between the model’s predicted values and actual observed results, calibration curves with 1000 bootstrap samples were further plotted. If the prediction curve is close to the ideal diagonal line, it indicates that the model has good calibration. Additionally, the goodness-of-fit was assessed using the Hosmer-Lemeshow test.

Moreover, patients were categorized into high- and low-risk subgroups based on the optimal cutoff value determined by the Youden index from the ROC curve of the prediction model. Differences in the incidence of postoperative hypoglycemia between the two subgroups were compared using the chi-square test or Fisher’s exact test.

No missing data were observed in the primary outcome (hypoglycemia). For the remaining variables, the proportion of missing data was below 5%; therefore, no form of data imputation was performed.

All analyses were performed using R software, version 4.5.2 (R Foundation for Statistical Computing, Vienna, Austria), and Statistical Product and Service Solutions, version 20.0 (SPSS Inc., Armonk, NY, USA). All *P* values were two-sided, and those < 0.05 were considered statistically significant.

## Results

### Patient characteristics

**Fig. 1:**
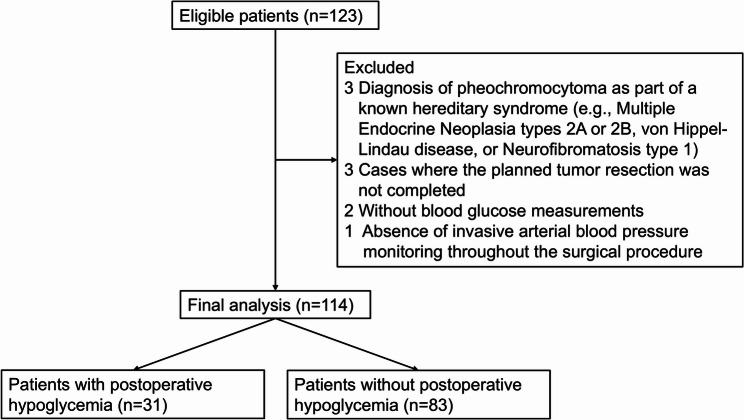
Recruitment flowchart

From September 1, 2019 to August 31, 2025, a total of 123 patients were screened for eligibility. Nine patients were excluded due to the following reasons: Diagnosis of pheochromocytoma as part of a known hereditary syndrome (e.g., Multiple Endocrine Neoplasia types 2A or 2B, von Hippel-Lindau disease, or Neurofibromatosis type 1); Absence of invasive arterial blood pressure monitoring throughout the surgical procedure; Cases where the planned tumor resection was not completed because of intraoperative complications, including massive hemorrhage, severe anaphylactic shock and acute heart failure; Incomplete or inaccessible perioperative blood glucose measurements. Finally, a total of 114 patients were enrolled into the final analysis (Fig. 1).

Among the 114 patients included in the study, 31 (27.2%) patients developed postoperative hypoglycemia. As summarized in Table 1, several baseline and intraoperative variables differed significantly between patients with and without postoperative hypoglycemia. Those who developed postoperative hypoglycemia were younger (54.0 [38.0–61.0] vs. 57.0 [48.0–67.0] years; *p* = 0.031) and had a lower BMI (20.9 [20.1–24.0] vs. 22.7 [20.8–25.3] kg/m²; *p* = 0.011). Additionally, patients with postoperative hypoglycemia exhibited higher preoperative plasma metanephrine level (226.0 [89.3–718.2] vs. 77.2 [38.0–310.0] pg/mL; *p* = 0.013). Elevated plasma metanephrine levels were observed in 83.9% of patients with hypoglycemia, which was much higher than that in patients without hypoglycemia (53.0%, *P* = 0.003). Patients with postoperative hypoglycemia were also accompanied with greater intraoperative hemodynamic fluctuations, reflected by elevated HR variability (11.9 ± 5.1 vs. 9.6 ± 3.7; *p* = 0.009), SBP variability (24.3 ± 7.7 vs. 20.6 ± 7.7; *p* = 0.023) and DBP variability (12.7 ± 4.1 vs. 10.5 ± 3.9; *p* = 0.010). The difference in ΔSBP (maximum systolic minus minimum systolic blood pressure) showed a trend toward significance (93.3 ± 29.4 vs. 82.0 ± 26.8; *p* = 0.057). The intraoperative use of alpha blockers and beta blockers was more common among patients who developed postoperative hypoglycemia (*p* = 0.025 and < 0.001, respectively). No significant differences were observed in other preoperative, laboratory, tumor-related or surgical related parameters.

**Table 1:** Baseline characteristics and main outcomes in patients who underwent pheochromocytoma resection

Variables	Total (*n* = 114)	Patients without hypoglycemia (*n* = 83)	Patients with hypoglycemia (*n* = 31)	*P* value
Age, median [IQR], yr	56.5 [43.8–65.3]	57.0 [48.0–67.0]	54.0 [38.0–61.0]	0.031
Female, *n* (%)	61(53.5)	41(49.4)	10 (64.5)	0.205
BMI, median [IQR], kg/m^2^	22.1 [20.6–25.0]	22.7 [20.8–25.3]	20.9 [20.1–24.0]	0.011
Maximal tumor diameter, median [IQR], cm	3.5 [2.5–4.9]	3.4 [2.4–4.8]	4.0 [2.6–5.2]	0.272
Tumor location, *n* (%)				0.547
Left adrenal gland	63 (55.3)	44 (53.0)	19 (61.3)	
Right adrenal gland	49 (43.0)	37 (44.6)	12 (38.7)	
Extra-adrenal gland (paraganglioma)	2 (1.8)	2 (2.4)	0 (0)	
Surgical resection technique, *n* (%)				0.952
Laparoscopic	96 (84.2)	70 (84.3)	26 (83.9)	
Robot-assisted	18 (15.8)	13 (15.7)	5 (16.1)	
Plasma metanephrine, median [IQR], pg/mL	120.9 [40.7–442.7]	77.2 [38.0–310.0]	226.0 [89.3–718.2]	0.013
Metanephrine elevation†, *n* (%)	70 (61.4)	44 (53.0)	26 (83.9)	0.003
Plasma normetanephrine, median [IQR], pg/mL	637.8 [272.1–1567.1]	580.3 [242.6–1464.8]	907.4 [437.2–2315.8]	0.121
Normetanephrine elevation‡, *n* (%)	95 (83.3)	67 (80.7)	28 (90.3)	0.221
Preoperative HCT, mean ± SD, %	39.9 ± 5.1	40.1 ± 5.0	39.6 ± 5.4	0.651
History of diabetes, *n* (%)	16 (14.0)	11 (13.3)	5 (16.1)	0.694
Preoperative glucose, median [IQR], mmol/L	5.6 [5.2–6.3]	5.6 [5.2–6.3]	5.6 [5.2–6.4]	0.547
Preoperative beta blocker use, *n* (%)	9 (7.9)	5 (6.0)	4 (12.9)	0.252
Preoperative alpha blocker use, *n* (%)	73 (64.0)	55 (66.3)	18 (58.1)	0.417
Surgical duration, median [IQR], min	99.0 [74.5–124.0]	99.0 [69.8–128.0]	99.0 [80.0–120.0]	0.857
Intraoperative blood loss, median [IQR], mL	50 [20–100]	50 [20–100]	50 [50–100]	0.284
Intraoperative fluid administration, median [IQR], mL	2000.0 [1637.5–2500.0]	2000.0 [1600.0–2500.0]	2000.0 [1950.0–2500.0]	0.910
Intraoperative urine output, median [IQR], mL	325.0 [200.0–500.0]	350.0 [200.0–500.0]	300.0 [200.0–400.0]	0.294
Preoperative SBP, mean ± SD, mmHg	128.9 ± 18.1	130.0 ± 18.0	125.8 ± 18.4	0.272
Preoperative DBP, mean ± SD, mmHg	78.2 ± 11.6	78.5 ± 12.2	77.3 ± 9.9	0.605
Preoperative HR, mean ± SD, bpm	74.8 ± 14.0	73.7 ± 13.2	77.7 ± 15.6	0.174
Hemodynamic parameters during surgery				
HR Variability, Mean ± SD	10.3 ± 4.2	9.6 ± 3.7	11.9 ± 5.1	0.009
SBP Variability, Mean ± SD	21.6 ± 7.8	20.6 ± 7.7	24.3 ± 7.7	0.023
DBP Variability, Mean ± SD	11.1 ± 4.0	10.5 ± 3.9	12.7 ± 4.1	0.010
ΔSBP*, Mean ± SD	85.0 ± 27.8	82.0 ± 26.8	93.3 ± 29.4	0.057
Alpha blocker use, *n* (%)	96 (84.2)	66 (79.5)	30 (96.8)	0.025
Vasopressor use, *n* (%)	70 (61.4)	49 (59.0)	21(67.7)	0.396
Beta blocker use, *n* (%)	45 (39.5)	24 (28.9)	21(67.7)	<0.001
ICU admission postoperatively, *n *(%)	8 (7.0)	6 (7.2)	2 (6.5)	0.885
Clinical outcomes				
Length of hospital stay after surgery, median [IQR], days	6.0 [5.0–7.0]	6.0 [4.0–7.0]	6.0 [6.0–8.0]	0.020
Hospitalization costs, median [IQR], Yuan	25748.0 [19841.3–35840.3]	23812.0 [19530.0–34602.0]	28110.0 [22183.0–40953.0]	0.084

*Abbreviations BMI *body mass index***,**** HCT* hematocrit, *SBP* systolic blood pressure, *DBP* diastolic blood pressure, *HR* heart rate, *ICU* intensive care unit, *IQR* interquartile range, *SD* standard deviation

† Metanephrine elevation is referred to plasma metanephrine level more than 62 pg/mL (above the upper limit of the normal range)

‡ Normetanephrine elevation is referred to plasma normetanephrine level more than 145 pg/mL (above the upper limit of the normal range)

*ΔSBP= maximum SBP - minimum SBP

### Independent predictors of hypoglycemia

Univariate analysis in Tables 1 and 2 revealed potential factors predicting postoperative hypoglycemia, including younger age, lower BMI, elevated preoperative plasma metanephrine, greater variability in SBP, DBP, and HR, intraoperative alpha blocker use and beta blocker use. All variance inflation factors were below 5, suggesting an absence of multicollinearity among the covariates (eTable 1 in the supplement). Furthermore, variables showing significance in univariate analysis were included in a multivariate logistic regression model to identify independent risk factors. As presented in Table 2, younger age (adjusted odds ratio 0.95, 95% CI 0.92 to 0.99; *p* = 0.015), preoperative metanephrine elevation (adjusted odds ratio 7.73, 95% CI 2.00 to 29.85; *p* = 0.003) and intraoperative beta blocker use (adjusted odds ratio 3.67, 95% CI 1.40 to 9.57; *p* = 0.008) remained independently associated with postoperative hypoglycemia.

**Table 2:** Univariate and multivariate logistic regression analyses of independent factors for postoperative hypoglycemia after pheochromocytoma resection

Variables	Univariable Analysis			Multivariable analysis		
	OR	95% CI	*P* value	OR	95% CI	*P* value
Age	0.97	0.94 to 0.99	0.025	0.95	0.92 to 0.99	0.015
BMI	0.83	0.72 to 0.96	0.014	-	-	-
Plasma metanephrine elevation	4.61	1.61 to 13.17	0.004	7.73	2.00 to 29.85	0.003
HR-SD	1.14	1.03 to 1.26	0.013	-	-	-
SBP-SD	1.06	1.01 to 1.12	0.030	-	-	-
DBP-SD	1.14	1.03 to 1.27	0.015	-	-	-
Intraoperative alpha blocker use	7.73	0.98 to 60.78	0.052	-	-	-
Intraoperative beta blocker use	5.16	2.12 to 12.57	< 0.001	3.67	1.40 to 9.57	0.008

### Development and performance of a predictive nomogram

**Fig. 2:**
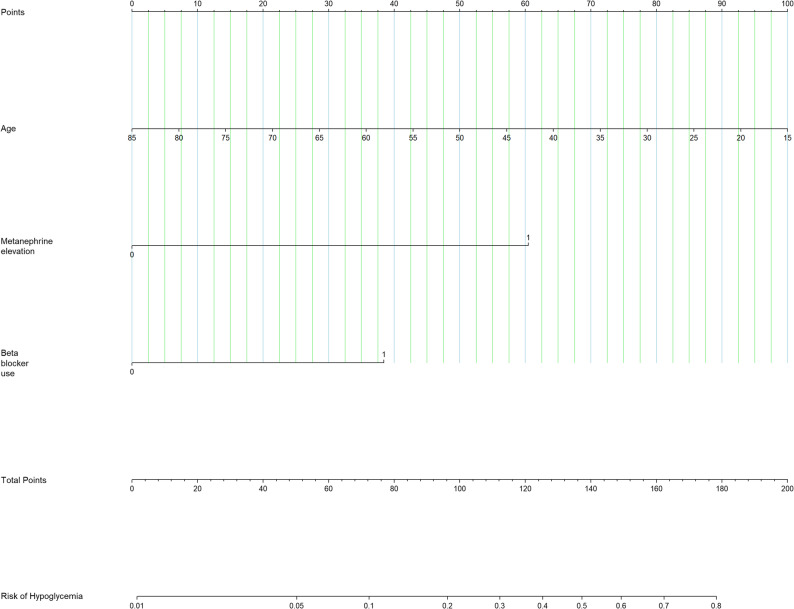
Nomogram for predicting postoperative hypoglycemia after pheochromocytoma resection

**Fig. 3:**
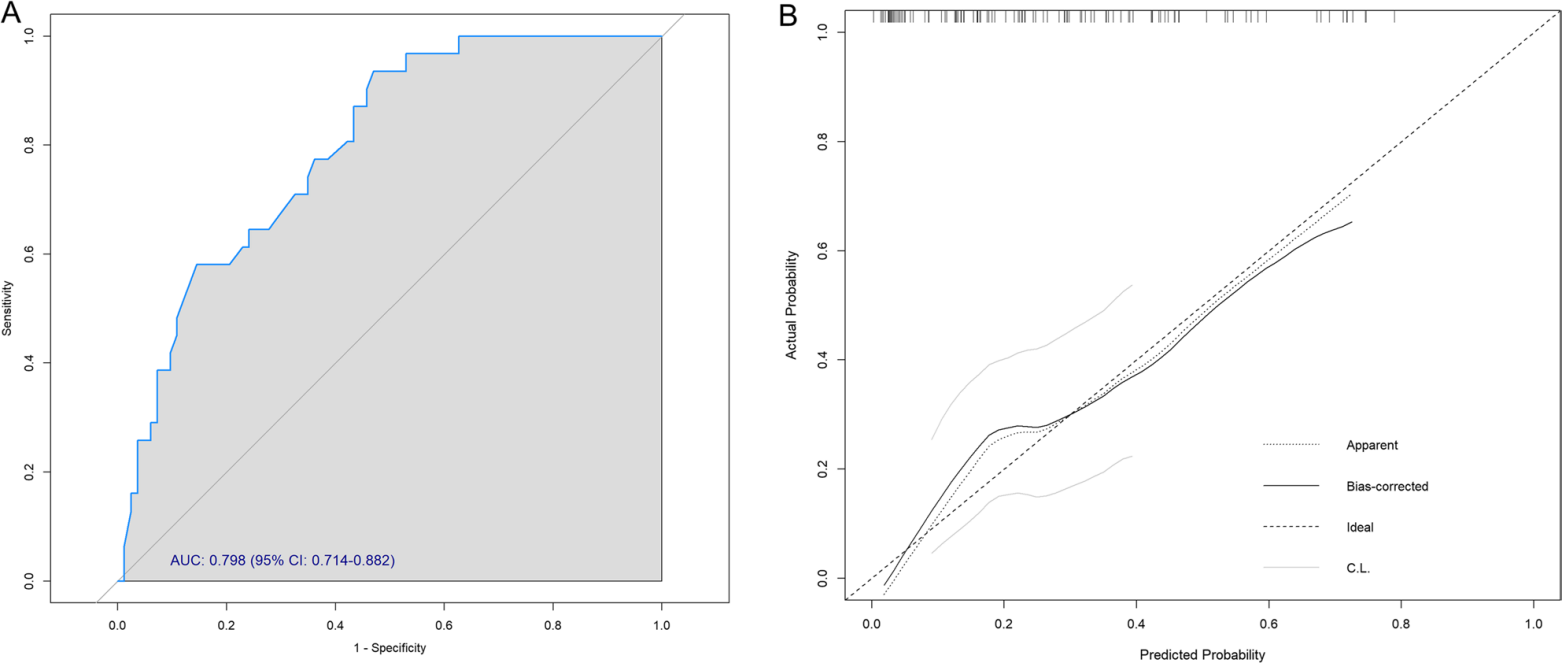
Discrimination (**A**) and calibration (**B**) analysis of the preoperative multivariable model

A nomogram was developed to estimate individualized risk of postoperative hypoglycemia based on the three independent predictors: age, preoperative metanephrine elevation and intraoperative beta blocker use (Fig. 2). Each variable contributes a point value, and the total points correspond to a predicted probability of hypoglycemia. The model demonstrated good discriminative ability, with an area under the ROC curve of 0.798 (95% CI: 0.714 to 0.882) (Fig. 3A). The internal validation of the prediction model was performed using the bootstrap method with 1,000 resamples, and a calibration curve was plotted (Fig. 3B). The results showed that the slope of the calibration curve was close to 1, and the predicted curve from the nomogram aligned well with the observed curve. This indicates good agreement between the predicted and actual risks of postoperative hypoglycemia, demonstrating satisfactory calibration of the model. Furthermore, the Hosmer–Lemeshow goodness-of-fit test also indicated that the model was well-calibrated (*p* = 0.720), suggesting no significant deviation between the predicted and observed hypoglycemia risks.

### Clinical risk stratification

Based on the highest Youden index, the optimal nomogram-predicted probability for predicting postoperative hypoglycemia was 0.16. Patients were then classified into two risk categories according to the nomogram-predicted probability: low-risk (≤ 0.16), and high-risk (> 0.16). The incidence of hypoglycemia was higher in high-risk patients than that in low-risk patients (low-risk: 4.3% vs. high-risk: 42.7%; absolute difference − 38.3%, 95% CI −53.7 to −22.9, *P* < 0.001) (Table 3).

**Table 3:** Comparison of incidence of hypoglycemia across subgroups

Variables	Total (*n* = 114)	Low risk (*n* = 46)	High risk (*n* = 68)	Absolute Difference	*P* value
Postoperative hypoglycemia, *n *(%)	31 (27.2)	2 (4.3)	29 (42.7)	−38.3 (−53.7 to −22.9)	< 0.001

## Discussion

The present study indicates that hypoglycemia was common in patients who underwent or in patients undergoing resection of pheochromocytomas, and was associated with delayed hospital stay. We further developed a multifactor model that integrates patient age, preoperative metanephrine elevation and intraoperative beta blocker use to offer a practical tool to immediately predict postoperative hypoglycemia. The model performed well in terms of discrimination and calibration in the cohort of patients undergoing pheochromocytoma resection.

Post-resection hypoglycemia represents a distinctive complication following the removal of pheochromocytoma. Reported incidence rates vary considerably across studies, ranging from 4.2% to 43% [8, 10]. In one perioperative analysis, 15% of patients developed hypoglycemia necessitating intensive glucose management, while other studies documented comparable rates of 13.3% when hypoglycemia was defined as serum glucose < 50 mg/dL, and 4.2% for levels < 55 mg/dL [19, 20]. A temporal analysis of 53 patients with pheochromocytomas undergoing surgery between 1996 and 2022 revealed a marked decline in hypoglycemia incidence after 2010 (0% vs. 28%, *P* = 0.003), suggesting potential improvements in perioperative management over time [21]. Our study further underscores the clinical significance of this phenomenon, revealing a high incidence of 27.2% and highlighting postoperative hypoglycemia as a frequent metabolic disturbance in this surgical cohort. We also found that the occurrence of postoperative hypoglycemia was associated with longer hospital stay after surgery and potential more hospital costs. These findings affirm that hypoglycemia is a common complication following pheochromocytoma resection and may worsen prognosis, which emphasizes the critical need for vigilant glucose monitoring after the procedure.

The established predictor for post-resection hypoglycemia is being critically re-evaluated due to its high incidence. Previous studies by Chen et al. and Araki et al. have independently identified preoperative 24-hour urinary epinephrine as a dominant independent predictor, with reporting a striking odds ratio of 19.8 [8, 10]. Similarly, our results confirm that elevated preoperative plasma metanephrine is strongly associated with and serve as an independent risk factor for postoperative hypoglycemia, aligning with and reinforcing the existing literature [8, 10]. In our cohort, 61.4% of patients had elevated plasma metanephrine, a proportion lower than that reported in a study of metastatic or progressive pheochromocytoma/paraganglioma (74% with elevated metanephrine or chromogranin A [22]), although absolute metanephrine concentrations were comparable [23].

The underlying mechanism likely relates to catecholamine physiology. Epinephrine promotes gluconeogenesis and glycogenolysis while inhibiting insulin secretion via β-adrenergic receptors [24]. This phenomenon has been demonstrated specifically in the pheochromocytoma population, and Wiesner et al. were able to show a reversal of insulin resistance in these patients after resection of the neoplasm [25]. Chronic epinephrine stimulation prior to surgery may lead to depletion of hepatic glycogen stores, impairing the body’s primary counter-regulatory response. Following tumor resection, the abrupt decline in circulating catecholamines triggers rebound hyperinsulinemia, which, coupled with a postoperative improvement in peripheral insulin sensitivity, significantly contributes to the development of hypoglycemia [20, 25–27].

Our study also identified intraoperative use of beta blockers as an independent predictor of postoperative hypoglycemia. Before pheochromocytoma resection, surgical manipulation often triggers further catecholamine release from the tumor, leading to tachycardia. In clinical settings, beta blocker administration is the common treatment for heart rate control before tumor resection [12]. The requirement for intraoperative beta blockade indirectly reflects a heightened state of adrenergic activity during surgery. This finding further corroborates our prior results that elevated epinephrine levels constitute a significant risk factor for postoperative hypoglycemia.

While previous studies have indicated that the dosage of alpha-adrenergic blockade is significantly associated with the occurrence of postoperative hypoglycemia [21], our results showed that alpha blocker use was not an independent predictor in the multivariate regression model. Furthermore, our study also did not identify preoperative normetanephrine levels as a predictor of hypoglycemia. These findings collectively suggest that beta-adrenergic receptors may play a more pivotal role than alpha-adrenergic mechanisms in the development of postoperative hypoglycemia following pheochromocytoma resection.

Variability in intraoperative heart rate and blood pressure was also examined in this study. Increased variability in hemodynamic parameters reflects heightened tumor activity and corresponds to greater fluctuations in circulating catecholamine levels before and after tumor resection [5]. This phenomenon occurs because surgical manipulation triggers a surge in catecholamine release, which stimulates cardiac activity. Following tumor resection, catecholamine secretion decreases abruptly, resulting in a dramatic fall in circulating levels. The combined effect of the pre-resection surge and the post-resection decline thereby leads to the pronounced fluctuation observed [28]. In our study, univariate analysis identified greater variability in heart rate, systolic blood pressure, and diastolic blood pressure as predictors of postoperative hypoglycemia, although these covariates were not retained in the final multivariate model. Higher variability of hemodynamic parameters observed in patients with postoperative hypoglycemia suggests that the magnitude of intraoperative catecholamine fluctuation may influence the likelihood of postoperative hypoglycemia.

Furthermore, we identified younger age as a novel and independent predictor of postoperative hypoglycemia. Younger patients are postulated to exhibit more robust pancreatic beta cell function and enhanced peripheral insulin sensitivity [29]. Consequently, the abrupt withdrawal of catecholamine-mediated adrenergic suppression of insulin secretion following tumor resection may unmask a state of relative hyperinsulinemia, precipitating a more profound and rapid decline in blood glucose [30]. Future studies featuring serial measurements of perioperative insulin and counter-regulatory hormones are warranted to definitively validate this age-dependent paradigm.

Secondary outcomes, including postoperative length of stay and hospital costs, were evaluated to compare the prognosis between patients with and without hypoglycemia after pheochromocytoma resection. Although a statistically significant difference in postoperative length of stay was observed between groups, the median values were similar, suggesting that the absolute difference may be of limited clinical relevance. This finding is consistent with a previous study, which also reported no clinically meaningful difference in length of stay in patients with and without hypoglycemia after pheochromocytoma resection (5 vs. 3 days, *P* = 0.10) [9]. Total hospital costs did not differ significantly between groups, although a numerical trend toward higher costs was observed in the hypoglycemia group. This lack of statistical significance may be related to the relatively small sample size. Overall, these findings suggest that postoperative hypoglycemia may be associated with modest differences in recovery metrics, while its impact on healthcare utilization remains uncertain. Further studies with larger sample sizes are warranted to better define its clinical and economic implications.

The primary innovation of our study lies in the successful translation of these physiological insights into a clinically actionable predictive tool. To our knowledge, this is the first study to establish a robust prediction model for pheochromocytoma related postoperative hypoglycemia that incorporates age, preoperative plasma metanephrine and intraoperative beta-blocker use. Our calibration curve indicated that the observed and predicted incidence of postoperative hypoglycemia were closely related, indicating that the newly constructed model was well calibrated. It was also confirmed that the newly constructed model did not have a significant Hosmer-Lemeshow test result. In addition, the AUC of our newly constructed model indicated good discrimination.

After risk stratification, postoperative hypoglycemia occurred more frequently in high-risk patients. This stepwise increase confirms the clinical utility of the nomogram in stratifying patient risk at the end of surgery. Among patients who developed postoperative hypoglycemia in our study, 51.5% required hypertonic glucose and 45.2% intensified monitoring, with no cases requiring ICU escalation for glycemic management (eTable 2). This finding suggests that ward-based management is sufficient for most patients and supports a resource-efficient, risk-stratified postoperative care pathway rather than routine ICU admission. For patients identified as high risk, intensified glucose monitoring with hourly blood glucose assessments during the first 24–48 h after surgery is recommended [31, 32], along with preemptive interventions when appropriate, including: (i) prophylactic glucose infusion—initiation of continuous intravenous dextrose (5–10% concentration) at rates of 20 mL/h immediately postoperatively, titrated to maintain blood glucose > 100 mg/dL (5.6 mmol/L); (ii) early nutritional support—prompt introduction of oral carbohydrate intake once hemodynamic stability permits; and (iii) ICU-level monitoring may be considered for selected patients with additional clinical concerns [31].

Several limitations of our study warrant consideration. First, the retrospective, single-center design inherently carries a risk of selection bias and unmeasured confounding. Although our sample size is substantial for this rare condition, it precluded adjustment for all potential confounders and more complex model development. External validation in a prospective, multi-institutional cohort is essential to confirm the generalizability of our findings. Second, given its retrospective design, postoperative blood glucose monitoring was not protocol-driven and lacked standardization in both timing and frequency. In routine clinical practice, glucose measurements were primarily obtained on the first postoperative day, with additional assessments performed only when clinically indicated. Moreover, the observation window for hypoglycemia was restricted to the first 24 postoperative hours in this study. These factors may have led to an underestimation of the true incidence of hypoglycemia. Prospective studies with standardized and extended glucose monitoring are warranted to better characterize postoperative hypoglycemia and to validate our predictive model. Third, the precise mechanistic link between catecholamines and insulin secretion remains to be fully elucidated; future research incorporating serial measurements of catecholamines, insulin, and counter-regulatory hormones is needed to delineate these pathways.

## Conclusions

Postoperative hypoglycemia is a common complication after resection of pheochromocytoma and associated with poor prognosis. We presented a nomogram including age, plasma metanephrine elevation and intraoperative beta blocker use to predict the risk for postoperative hypoglycemia. This approach holds the promise of improving patient safety and outcomes by enabling proactive management of a common and dangerous complication in a high-risk surgical population.

## Supplementary Information

Supplementary Material 1.

## Data Availability

Data supporting the findings of this study are held by the First Affiliated Hospital, Zhejiang University School of Medicine and are subject to restricted access due to licensing agreements related to the current study. They are not publicly available but may be requested from the corresponding author upon reasonable request and approval from the hospital.

## Abbreviations

STROBE: Strengthening the reporting of observational studies in epidemiology
SBP: Systolic blood pressure
DBP: Diastolic blood pressure
HR: Heart rate
BMI: Body mass index
SD: Standard deviation
IQR: Interquartile range
OR: Odds ratio
CI: Confidence interval
ROC: Receiver operating characteristic curve
AUC: Area under the receiver operating characteristic curve

## References

[1] Vitturi G, Crisafulli S, Alessi Y, et al. Global epidemiology of pheochromocytoma: a systematic review and meta-analysis of observational studies. J Endocrinol Invest. 2025;48(12):2813–25. 10.1007/s40618-025-02639-9. 40601232 10.1007/s40618-025-02639-9

[2] Neumann HPH, Longo DL, Young WF, Eng C. Pheochromocytoma and Paraganglioma. N Engl J Med. 2019;381(6):552–65. 10.1056/NEJMra1806651. 31390501 10.1056/NEJMra1806651

[3] Lenders JWM, Duh Q-Y, Eisenhofer G, et al. Pheochromocytoma and Paraganglioma: An Endocrine Society Clinical Practice Guideline. J Clin Endocrinol Metabolism. 2014;99(6):1915–42. 10.1210/jc.2014-1498. 10.1210/jc.2014-1498 24893135

[4] Carvalho IC, Machado MVB, Morais JP, et al. The role of the adrenalectomy in the management of pheochromocytoma: the experience of a Portuguese referral center. Endocrine. 2024;86(1):409–16. 10.1007/s12020-024-03916-y. 38849646 10.1007/s12020-024-03916-y PMC11445341

[5] Ando Y, Ono Y, Sano A, et al. Clinical characteristics and outcomes of pheochromocytoma crisis: a literature review of 200 cases. J Endocrinol Invest. 2022;45(12):2313–28. 10.1007/s40618-022-01868-6. 35857218 10.1007/s40618-022-01868-6

[6] Araujo-Castro M, Herrera A, Wang Y, et al. Postoperative Outcomes in Normotensive and Hypertensive Pheochromocytomas: An International Study. J Clin Endocrinol Metabolism. 2025;110(11):e3719–29. 10.1210/clinem/dgaf154. 10.1210/clinem/dgaf154 PMC12527436 40048695

[7] Nazari MA, Hasan R, Haigney M, et al. Catecholamine-induced hypertensive crises: current insights and management. Lancet Diabetes Endocrinol. 2023;11(12):942–54. 10.1016/s2213-8587(23)00256-5. 37944546 10.1016/S2213-8587(23)00256-5

[8] Araki S, Kijima T, Waseda Y, et al. Incidence and predictive factors of hypoglycemia after pheochromocytoma resection. Int J Urol. 2018;26(2):273–7. 10.1111/iju.13864. 30467902 10.1111/iju.13864

[9] Chen Y, Hodin RA, Pandolfi C, Ruan DT, McKenzie TJ. Hypoglycemia after resection of pheochromocytoma. Surgery. 2014;156(6):1404–8. 10.1016/j.surg.2014.08.020. discussion 1408–1409. 25456920 10.1016/j.surg.2014.08.020

[10] Chen Y, Hodin RA, Pandolfi C, Ruan DT, McKenzie TJ. Hypoglycemia after resection of pheochromocytoma. Surgery. 2014;156(6):1404–9. 10.1016/j.surg.2014.08.020. 25456920 10.1016/j.surg.2014.08.020

[11] De Leo A, Vara G, Paccapelo A, et al. Computerized tomography texture analysis of pheochromocytoma: relationship with hormonal and histopathological data. J Endocrinol Invest. 2022;45(10):1935–44. 10.1007/s40618-022-01826-2. 35680695 10.1007/s40618-022-01826-2 PMC9463266

[12] Gombert AJ, Nerantzinis AM, Li J, et al. The Perioperative Biochemical and Clinical Considerations of Pheochromocytoma Management. Int J Mol Sci. 2025;26(13). 10.3390/ijms26136080. 10.3390/ijms26136080 PMC12250190 40649858

[13] Cohen JB, Abu Salman L, Bennett BJ, Cohen DL. Ambulatory blood pressure monitoring before and after resection of catecholamine-secreting pheochromocytoma or paraganglioma. J Hum Hypertens. 2025;39(5):369–75. 10.1038/s41371-025-01008-6. 40140505 10.1038/s41371-025-01008-6 PMC12069104

[14] Shen X, Tao H, Chen W, et al. Perioperative blood pressure variability as a risk factor for postoperative delirium in the patients receiving cardiac surgery. BMC Anesthesiol. 2024;24(1). 10.1186/s12871-024-02817-x. 10.1186/s12871-024-02817-x PMC11587544 39581994

[15] Yao H, Yuan S, Pan H, et al. Predicting hypoglycemia risk after gastrointestinal surgery in type 2 diabetes mellitus: a retrospective cohort study. Front Endocrinol 16. 2025. 10.3389/fendo.2025.1590780. 10.3389/fendo.2025.1590780 PMC12277139 40692592

[16] Jacobi J, Bircher N, Krinsley J, et al. Guidelines for the use of an insulin infusion for the management of hyperglycemia in critically ill patients. Crit Care Med. 2012;40(12):3251–76. 10.1097/CCM.0b013e3182653269. 23164767 10.1097/CCM.0b013e3182653269

[17] Araki S, Kijima T, Waseda Y, et al. Incidence and predictive factors of hypoglycemia after pheochromocytoma resection. Int J Urol. 2019;26(2):273–7. 10.1111/iju.13864. 30467902 10.1111/iju.13864

[18] Peduzzi P, Concato J, Kemper E, Holford TR, Feinstein AR. A simulation study of the number of events per variable in logistic regression analysis. J Clin Epidemiol. 1996;49(12):1373–9. 10.1016/s0895-4356(96)00236-3. 8970487 10.1016/s0895-4356(96)00236-3

[19] Plouin PF, Duclos JM, Soppelsa F, Boublil G, Chatellier G. Factors associated with perioperative morbidity and mortality in patients with pheochromocytoma: analysis of 165 operations at a single center. J Clin Endocrinol Metab. 2001;86(4):1480–6. 10.1210/jcem.86.4.7392. 11297571 10.1210/jcem.86.4.7392

[20] Akiba M, Kodama T, Ito Y, Obara T, Fujimoto Y. Hypoglycemia induced by excessive rebound secretion of insulin after removal of pheochromocytoma. World J Surg. 1990;14(3):317–24. 10.1007/BF01658514. 2195784 10.1007/BF01658514

[21] Yamanashi Y, Yoshida Y, Nakai T, et al. The frequency of postoperative hypoglycemia after pheochromocytoma surgery is decreasing. World J Surg. 2024;48(12):2918–24. 10.1002/wjs.12368. 39384339 10.1002/wjs.12368 PMC11619746

[22] Baudin E, Goichot B, Berruti A, et al. Sunitinib for metastatic progressive phaeochromocytomas and paragangliomas: results from FIRSTMAPPP, an academic, multicentre, international, randomised, placebo-controlled, double-blind, phase 2 trial. Lancet. 2024;403(10431):1061–70. 10.1016/S0140-6736(23)02554-0. 38402886 10.1016/S0140-6736(23)02554-0

[23] Deutschbein T, Unger N, Jaeger A, et al. Influence of various confounding variables and storage conditions on metanephrine and normetanephrine levels in plasma. Clin Endocrinol (Oxf). 2010;73(2):153–60. 10.1111/j.1365-2265.2009.03761.x. 20039892 10.1111/j.1365-2265.2009.03761.x

[24] Deibert DC, DeFronzo RA. Epinephrine-induced insulin resistance in man. J Clin Invest. 1980;65(3):717–21. 10.1172/JCI109718. 6243677 10.1172/JCI109718 PMC371414

[25] Wiesner TD, Bluher M, Windgassen M, Paschke R. Improvement of insulin sensitivity after adrenalectomy in patients with pheochromocytoma. J Clin Endocrinol Metab. 2003;88(8):3632–6. 10.1210/jc.2003-030000. 12915647 10.1210/jc.2003-030000

[26] Bluher M, Windgassen M, Paschke R. Improvement of insulin sensitivity after adrenalectomy in patients with pheochromocytoma. Diabetes Care. 2000;23(10):1591–2. 10.2337/diacare.23.10.1591. 11023156 10.2337/diacare.23.10.1591

[27] Reynolds C, Wilkins GE, Schmidt N, Doll WA, Blix PM. Hyperinsulinism after removal of a pheochromocytoma. Can Med Assoc J. 1983;129(4):349–53. 6871802 PMC1875123

[28] Tadic M, Sala C, Carugo S, Cuspidi C. Effect of surgical treatment on myocardial strain in patients with pheochromocytoma and paraganglioma: a mini-review and meta-analysis. J Endocrinol Invest. 2021;44(11):2327–32. 10.1007/s40618-021-01631-3. 34235707 10.1007/s40618-021-01631-3

[29] Lopez C, Bima C, Bollati M, et al. Pathophysiology and Management of Glycemic Alterations before and after Surgery for Pheochromocytoma and Paraganglioma. Int J Mol Sci. 2023;24(6). 10.3390/ijms24065153. 10.3390/ijms24065153 PMC10049717 36982228

[30] Khatiwada S, Agarwal S, Kandasamy D, et al. Diabetes mellitus in pheochromocytoma and paraganglioma: Prevalence, dynamics of insulin secretion / sensitivity and predictors of remission. Diabetes Metabolic Syndrome: Clin Res Reviews. 2020;14(6):2169–75. 10.1016/j.dsx.2020.10.030. 10.1016/j.dsx.2020.10.030 33395777

[31] Jinjing W, Kang C, Xufei L, et al. Chinese clinical practice guidelines for perioperative blood glucose management. Diabetes Metab Res Rev. 2021;37(7):e3439. 10.1002/dmrr.3439. 33605539 10.1002/dmrr.3439

[32] Lenders JW, Duh QY, Eisenhofer G, et al. Pheochromocytoma and paraganglioma: an endocrine society clinical practice guideline. J Clin Endocrinol Metab. 2014;99(6):1915–42. 10.1210/jc.2014-1498. 24893135 10.1210/jc.2014-1498

