# Supplementary material for: Prediction of postoperative hypoglycemia in patients with pheochromocytoma resection: a retrospective study
Source: BMC Surg. 2026 Apr 15;26:364. doi: 10.1186/s12893-026-03734-1 (PMC13220516; doi:10.1186/s12893-026-03734-1)
Supplement: Supplementary file 1 — Supplementary Material 1. [file 12893_2026_3734_MOESM1_ESM.docx]

Online Supplement

eTable 1 Collinearity assessment between baseline characteristics and postoperative hypoglycemia

| Variables | VIF |
| --- | --- |
| Age | 1.598 |
| BMI | 1.172 |
| Metanephrine abnormal | 1.241 |
| HR-SD | 1.196 |
| SBP-SD | 3.140 |
| DBP-SD | 3.036 |
| Intraoperative antihypertensive use | 1.287 |
| Intraoperative β-blocker use | 1.457 |

eTable 2 The details of intensive glucose management in patients with postoperative hypoglycemia

| Management | No. of Patients |
| --- | --- |
| Intravenous administration of hypertonic glucose solution (50% dextrose) for documented hypoglycemia | 16 (51.5%) |
| Intensified hemodynamic and metabolic monitoring, including hourly blood glucose assessments until stable euglycemia was achieved | 14 (45.2%) |
| Escalation of care to ICU for refractory cases requiring advanced hemodynamic support or continuous glucose monitoring | 0 |
